# Dose–volume constraints for head‐and‐neck cancer in carbon ion radiotherapy: A literature review

**DOI:** 10.1002/cam4.5641

**Published:** 2023-02-17

**Authors:** Maria Varnava, Atsushi Musha, Mutsumi Tashiro, Nobuteru Kubo, Naoko Okano, Hidemasa Kawamura, Tatsuya Ohno

**Affiliations:** ^1^ Gunma University Heavy Ion Medical Center Maebashi Gunma Japan; ^2^ Department of Oral and Maxillofacial Surgery and Plastic Surgery Gunma University Graduate School of Medicine Maebashi Gunma Japan; ^3^ Department of Radiation Oncology Gunma University Graduate School of Medicine Maebashi Gunma Japan

**Keywords:** carbon ion radiotherapy, dose–volume constraints, head‐and‐neck cancer, organs at risk

## Abstract

**Background:**

Carbon ion radiotherapy (CIRT) has been applied in cancer treatment for over 25 years. However, guidelines for dose–volume constraints have not been established yet. The aim of this review is to summarize the dose–volume constraints in CIRT for head‐and‐neck (HN) cancer that were determined through previous clinical studies based on the Japanese models for relative biological effectiveness (RBE).

**Methods:**

A literature review was conducted to identify all constraints determined for HN cancer CIRT that are based on the Japanese RBE models.

**Results:**

Dose–volume constraints are reported for 17 organs at risk (OARs), including the brainstem, ocular structures, masticatory muscles, and skin. Various treatment planning strategies are also presented for reducing the dose delivered to OARs.

**Conclusions:**

The reported constraints will provide assistance during treatment planning to ensure that radiation to OARs is minimized, and thus adverse effects are reduced. Although the constraints are given based on the Japanese RBE models, applying the necessary conversion factors will potentially enable their application by institutions worldwide that use the local effect model for RBE.

## Graphical abstract

This study summarizes the dose–volume constraints for head and neck cancer in carbon ion radiotherapy that are based on the Japanese models for relative biological effectiveness. The constraints are divided into hard and soft to help in deciding which constraints to prioritize during plan optimization. Applying the necessary conversion factors will potentially enable their application by institutions that use the local effect model.

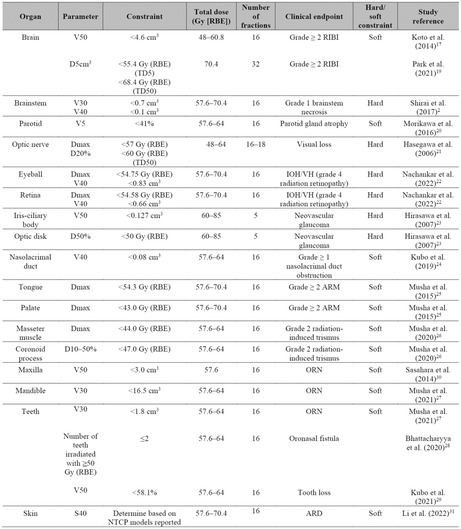

## 1 INTRODUCTION

Head‐and‐neck (HN) tumors are ranked as the sixth most common malignancy worldwide. Treatment options depend on the stage, anatomical site, and surgical accessibility of the HN tumors.^1^ Depending on the stage, squamous cell carcinomas (SCCs) can be treated with surgery, radiotherapy, or a combination of surgery, radiotherapy, and/or chemotherapy. On the other hand, non‐SCCs, being usually resistant to chemotherapy and radiotherapy, have limited treatment options.^2^

Carbon ion radiotherapy (CIRT) has various advantages compared with X‐ray radiotherapy (XRT). It has higher biological effectiveness than XRT and has already shown promising results for the treatment of resistant tumors like sarcoma, adenocarcinoma, and other non‐SCCs.^3^, ^4^, ^5^ Furthermore, because of its characteristic Bragg peak, it can deliver a localized high dose to the target while minimizing the dose delivered to organs at risk (OARs).^4^, ^5^ The beam delivery technique and the distance between the target and OARs dictate the degree of complications and whether tumor control can be achieved. Therefore, it is important to establish constraints based on dose–volume histogram (DVH) parameters so that treatment plans can be optimized for each patient.

DVH parameters have been considered predictive factors for adverse effects in various organs. Such parameters are being used as constraints during treatment planning to ensure OAR complications are minimized. In XRT, constraints have been established based on the empirical clinical practice of radiotherapy institutions. Emami et al. suggested dose–volume parameters associated with a given risk of normal tissue complications.^6^ Since constraints have to be validated by long‐term clinical toxicity data, a comprehensive review was published in 2010, which provided an update on the previously suggested constraints, as part of the Quantitative Analysis of Normal Tissue Effects in Clinic initiative.^7^ With the evolution of XRT, constraints have also evolved from simple numeric values to more complex DVH constraints in order to meet the needs of current radiotherapy techniques.^8^

Particle therapy is biologically and qualitatively different from XRT. International Commission on Radiation Units and Measurements and clinical practice recommend the use of relative biological effectiveness (RBE)‐weighted doses in particle therapy.^9^ In proton therapy, the radiobiological properties of the proton are described with a constant scaling of 1.1.^10^ This implies that the same constraints as X‐ray intensity‐modulated radiotherapy (IMRT) can be used for proton therapy.^11^

In CIRT, the RBE models are more complex compared with the constant scaling factor used in proton therapy. Currently, three RBE models are used in clinical practice: the mixed‐beam model, the modified microdosimetric kinetic model (MKM), which is an update of the mixed‐beam model, and the local effect model (LEM).^12^, ^13^, ^14^, ^15^ The mixed‐beam model and MKM are performed at Japanese institutions, with the MKM being more widely used. The LEM is used at European and Chinese institutions. The models are different with regard to their physical and mathematical assumptions and consider different endpoints.^11^ Recent articles have already reported LEM‐based dose–volume constraints that are currently being used at European facilities.^11^, ^16^ One study also reported constraints based on the Japanese RBE models; however, the complications considered were very few.^11^

Previous studies have associated various constraints with OAR complications related to the HN cancer CIRT.^2^, ^17^, ^18^, ^19^, ^20^, ^21^, ^22^, ^23^, ^24^, ^25^, ^26^, ^27^, ^28^, ^29^, ^30^, ^31^ This review provides an extensive list of constraints for HN cancer CIRT based on the Japanese RBE models. Any lacking information required to form a thorough list of constraints for all OARs related to HN cancer is also pointed out. This review will act as an assisting guide for treatment planning and be the first step in establishing constraints in CIRT for HN cancer, which would play an important role in improving CIRT implementation.

In the following sections, dose–volume constraints for OARs will be introduced. The CIRT dose is expressed in units of Gy (RBE), defined as the product of the physical dose and carbon ion RBE. The RBE is evaluated using the biological NIRS model, which is based on the linear‐quadratic model.^32^

## 2 BRAIN

Radiation‐induced brain injury (RIBI) is a serious complication. It can be classified as acute, early delayed, and late reaction, with the latter being one of the most serious complications.^17^, ^18^

Koto et al. investigated the occurrence of RIBI in 47 patients with skull base tumors treated using a total dose of 48–60.8 Gy (RBE) delivered in 16 fractions.^17^ RIBI severity was evaluated based on magnetic resonance imaging findings using the late effects of normal tissue‐subjective, objective, management, and analytic scales criteria. Clinical symptoms were graded based on the Radiation Therapy Oncology Group/European Organization for Research and Treatment of Cancer Late Radiation Morbidity Scoring System (RTOG/EORTC). Univariate analysis showed that V40 (volume receiving 40 Gy [RBE]) ≥ 7.6 cm^3^ and V50 ≥ 4.6 cm^3^ were correlated with the development of grade ≥2 RIBI. Among these parameters, V50 was shown to be an independent risk factor for the occurrence of grade ≥2 RIBI.^17^ The 5‐year occurrence rates for grade ≥2 RIBI for V50 ≥ 4.6 cm^3^ and V50 < 4.6 cm^3^ were 34.3% and 15.6%, respectively. In most cases, grade ≥2 RIBI developed from within the 40 Gy (RBE) isodose line. This agrees with a previous study, which reported that the average dose at which RIBI initially occurred was 50 Gy (RBE) for 40 patients that developed RIBI.^18^

A recent study also investigated the RIBI occurrence in CIRT, which was evaluated using the Common Terminology Criteria for Adverse Effects (CTCAE).^19^ Data from 104 patients with HN and skull base tumors who received 70.4 Gy (RBE) in 32 fractions were analyzed. Univariate analysis revealed that 84 out of the 94 DVH parameters investigated were significantly correlated with grade ≥2 RIBI occurrence. From these parameters, the dose covering 5 cm^3^ (D5cm^3^) of the brain was selected for the multivariate analysis based on the Akaike information criterion value to avoid multicollinearity. Multivariate analysis showed that D5cm^3^ is a significant risk factor. Tolerance doses for D5cm^3^ were estimated for the 5% (TD5) and 50% (TD50) probabilities of developing grade ≥2 RIBI: D5cm^3^ < 55.4 Gy (RBE) and D5cm^3^ < 68.4 Gy (RBE), respectively.

## 3 BRAINSTEM

Similar to RIBI, radiation‐induced brainstem necrosis is a serious adverse effect and may be life‐threatening.^2^ It is a rare event that generally develops a few years after treatment. Brainstem necrosis can be the reason for various neurological symptoms depending on the anatomical region of the necrosis. This is because the brainstem has important roles in controlling the cranial nerves, cardiac function, and respiratory motion.^2^ Therefore, it is necessary to consider the brainstem as a separate entity from the brain and assign more conservative constraints.

To achieve this, Shirai et al. investigated risk factors for asymptomatic grade 1 brainstem necrosis.^2^ Data from 62 HN cancer patients, who were treated using 57.6–70.4 Gy (RBE) in 16 fractions, were analyzed. Brainstem necrosis was graded according to the CTCAE. Receiver operating characteristic (ROC) analysis revealed significant cut‐off values for various DVH parameters: maximum dose (Dmax) < 48 Gy (RBE), D1cm^3^ < 27 Gy (RBE), V40 < 0.1 cm^3^, V30 < 0.7 cm^3^, and V20 < 1.4 cm^3^. Multivariate analysis showed that V30 and V40 are independent risk factors for brainstem necrosis, with the V30 being the most significant risk factor. The 2‐year grade 1 brainstem necrosis rate for V30 ≥ 0.7 cm^3^ and V30 < 0.7 cm^3^ were 33% and 0%, respectively. The 2‐year grade 1 brainstem necrosis rate for V40 ≥ 0.1 cm^3^ and V40 < 0.1 cm^3^ were 40% and 0%, respectively.

## 4 PAROTID GLANDS

Xerostomia is a critical complication, which may lead to various adverse effects that burden the patient, such as dysphagia, dysgeusia, articulatory disorders, periodontal disease, and respiratory infection.^20^

In CIRT, severe xerostomia is not a common effect because the dose delivered to the salivary glands can be reduced. Contrary to XRT, in which case both parotid glands are irradiated as part of the prophylactic irradiation of lymph nodes, both parotids are rarely irradiated in CIRT. One study observed cases of mild xerostomia development after CIRT and implied that xerostomia might be associated with parotid gland atrophy.^20^ The study investigated the relationship between DVH parameters and parotid gland atrophy for 54 patients treated with 57.6 or 64 Gy (RBE) in 16 fractions, whose parotid glands were irradiated during CIRT. Radiation‐induced xerostomia was evaluated based on the RTOG/EORTC. The V5–V20, mean dose (Dmean), and Dmax indices were significant prognostic factors of parotid gland volume decrease, indicating parotid gland atrophy. Multivariate analysis, though, identified only V5 as a prognostic factor for parotid gland volume decrease. The mean V5 values corresponding to patients with parotid gland volume decrease and non‐volume decrease were V5 = 65.4% and V5 = 41.0%, respectively. Since no value for the V5 constraint was given for preventing parotid gland atrophy, we suggest the use of V5 < 41% as a constraint. However, further research is required to obtain an accurate value for the V5 constraint.

Salivary flow reduction is another complication following parotid gland irradiation that causes xerostomia.^33^ Compared with parotid gland atrophy, salivary flow reduction may be a more important endpoint because it is related to the function of the parotids. Constraints for the salivary flow reduction after CIRT should also be determined.

## 5 OCULAR STRUCTURES: OPTIC NERVE, EYEBALL, RETINA, IRIS‐CILIARY BODY, AND OPTIC DISK

The irradiation of ocular structures could result in serious complications, such as visual loss and neovascular glaucoma, which may lead to enucleation.^21^, ^22^, ^23^

One of the causes of visual loss is optic nerve irradiation.^21^ A study investigated the tolerance dose for retention of visual acuity in 30 patients who were treated with a total dose of 48–64 Gy (RBE) in 16–18 fractions.^21^ Tolerance doses were analyzed for a total of 54 optic nerves that were included in the irradiated volume. Multivariate analysis revealed that the optic nerve D20% is a significant prognostic factor. Based on the normal tissue complication probability (NTCP) curve constructed for visual loss, it was determined that 10% and 50% probability of visual loss are associated with D20% = 40 Gy (RBE) and D20% = 60 Gy (RBE), respectively. Furthermore, based on the findings, it was suggested that Dmax should be kept at less than 57 Gy (RBE). The 35 optic nerves irradiated with <57 Gy (RBE) resulted in no visual loss. However, 11 of the 19 optic nerves irradiated with >57 Gy (RBE) resulted in a decrease in visual acuity; for the optic nerve Dmax = 57–65 Gy (RBE), the incidence of visual loss was 45%, while for Dmax > 65 Gy (RBE), the incidence of visual loss was 75%. The D20% parameter was reported to be more accurate than the Dmax parameter because there were cases in which Dmax fluctuated due to region‐of‐interest delineation, the use of the patch field technique, and positioning accuracy.^21^ The study recommends using only D20% < 60 Gy (RBE) as a constraint, however Dmax < 57 Gy (RBE) could be used as a reference during treatment planning.

Eye irradiation can cause intraocular hemorrhage/vitreous hemorrhage (IOH/VH), which could manifest as a haze or sudden painless visual loss due to the presence of blood in the vitreous humor.^22^ Nachankar et al. examined the association between DVH parameters of the eyeball, retina, and optic nerve and the IOH/VH occurrence in 79 patients with non‐SCC HN cancers.^22^ The patients were treated mainly with 64–70.4 Gy (RBE) in 16 fractions or 57.6 Gy (RBE) in cases of major skin or mucosal involvement. IOH/VH occurrence was defined as the hyperintense signal in vitreous humor on T_1_‐weighted images. Univariate analysis revealed that all parameters investigated (eyeball/retina/optic nerve Dmax and V10–60), except the optic nerve Dmax and V60, are significant predictors of IOH/VH. By considering the fact that ocular structures are serial and parallel organs, the study suggests the use of both Dmax and volumetric parameters. From the volumetric parameters, the use of V40 is suggested because of its clinical relevance; according to the treatment protocol, the planning target volume (PTV) changes after an initial irradiation of 36 Gy (RBE). Furthermore, the V40 parameter for the eyeball and retina had better accuracy in classifying the development or non‐development of IOH/VH than the optic nerve V40. Therefore, the study proposes the use of the eyeball and retina Dmax and V40. The cut‐off values for the eyeball Dmax, eyeball V40, retina Dmax, and retina V40 were 54.75 Gy (RBE), 0.83 cm^3^, 54.58 Gy (RBE), and 0.66 cm^3^, respectively. The 5‐year cumulative incidence rates for each parameter were 23.3% versus 0% (eyeball Dmax ≥ 54.75 vs. < 54.75 Gy [RBE]), 22.2% versus 1.12% (eyeball V40 ≥ 0.83 vs. < 0.83 cm^3^), 20.69% versus 1.15% (retina Dmax ≥ 54.58 vs. < 54.58 Gy [RBE]), and 22.22% versus 1.12% (retina V40 ≥ 0.66 vs. < 0.66 cm^3^).

Neovascular glaucoma is one of the complications after radiotherapy for choroidal melanoma, and its occurrence may indicate whether the eye can be preserved after treatment.^23^ Hirasawa et al. analyzed data from 55 patients diagnosed with choroidal melanoma located near the optic disk.^23^ The patients underwent CIRT with a total dose of 60–85 Gy (RBE) delivered in 5 fractions. The V50 of the iris‐ciliary body, V50 of the eyeball wall, which was used as a substitute for the retina, and optic disk irradiation (ODI) were considered as possible prognostic factors of neovascular glaucoma occurrence. ODI was defined as the irradiation of >50 Gy (RBE) to at least 50% volume of the optic disk. Multivariate analysis identified as independent significant predictors the iris‐ciliary body V50 (V50 ≥ 0.127 cm^3^) and ODI. The 3‐year neovascular glaucoma occurrence rates for the iris‐ciliary body V50 ≥ 0.127 cm^3^ and V50 < 0.127 cm^3^ were 71.4% and 11.5%, respectively. The respective rates for ODI and no ODI were 62.9% and 28.4%, respectively.

## 6 NASOLACRIMAL DUCT

Nasolacrimal duct obstruction is a common complication in HN carcinoma patients, but it does not affect patient survival.^24^ Nevertheless, it affects the quality of life (QOL) of the patient, particularly one of its symptoms called epiphora.

Kubo et al. investigated the correlation between dose–volume parameters and the incidence of nasolacrimal duct obstruction in 28 patients treated for HN non‐SCCs.^24^ Prescription dose was set at 57.6 or 64 Gy (RBE) delivered in 16 fractions. Nasolacrimal duct obstruction was evaluated based on the CTCAE. Dose–volume analysis showed significant differences in irradiated nasolacrimal duct volumes (V10–60) between patients who experienced nasolacrimal duct obstruction and patients who did not. Significant differences were also observed in the nasolacrimal duct Dmax and Dmean. ROC analysis revealed cut‐off values for nasolacrimal duct obstruction with an accuracy of >96%: V10 ≥ 0.24 cm^3^, V20 ≥ 0.24 cm^3^, V30 ≥ 0.17 cm^3^, V40 ≥ 0.08 cm^3^, V50 ≥ 0.19 cm^3^, and V60 ≥ 0.04 cm^3^. Similar to Nachankar et al.,^22^ Kubo et al.^24^ also suggest the use of V40 because of its clinical applicability. The univariate analysis confirmed that V40 is a significant risk factor for nasolacrimal duct obstruction. The 5‐year cumulative incidence of nasolacrimal obstruction for patients with V40 ≥ 0.08 cm^3^ was 93.8%, whereas for patients with V40 < 0.08 cm^3^ was 0%. Furthermore, a higher incidence of nasolacrimal duct obstruction was observed for patients who had a tumor in the nasal cavity or maxillary sinus (93.3%) than for patients who had a tumor at other anatomical sites.

## 7 ORAL MUCOSA

Acute radiation mucositis (ARM) is one of the major toxicities of radiotherapy.^34^ It may cause oral pain and dysphagia, thus affecting the QOL, and could be life‐threatening. Musha et al. created an oral mucosal dose surface model for the tongue and palate, which was used to investigate the correlation of DVH parameters with the severity of ARM.^25^ Thirty‐nine HN cancer patients were treated with a total dose of 57.6, 64, or 70.4 Gy (RBE) in 16 fractions. ARM was evaluated using both the CTCAE and RTOG criteria. A dose–response relationship was observed between Dmax and the ARM grade assessed using the RTOG criteria, whereas no relationship was observed when the CTCAE was considered. This indicates that the RTOG criteria might be a better choice in the objective evaluation of ARM. For the incidence of grade ≥2 ARM, ROC analysis showed cut‐off values of Dmax equal to 43.0 and 54.3 Gy (RBE) for the palate and tongue, respectively.

Besides Dmax, significant differences were also observed in the V5–V40 parameters between grades 0–1 and grades 2–3. This indicates that low‐to‐medium radiation doses may promote grade 2–3 ARM development. Further research is necessary to determine any additional parameters associated with ARM occurrence.

## 8 MASTICATORY MUSCLES

Radiation‐induced trismus is one of the late adverse effects in HN cancer patients undergoing radiotherapy, particularly in patients with tumors near the temporomandibular joint. Trismus causes restricted mouth opening, which leads to difficulty in mastication, speech, dental hygiene maintenance, and possible malnutrition, thus affecting QOL.^35^, ^36^

One study investigated the relationship between trismus occurrence and incident dose in 31 patients treated with 57.6 or 64 Gy (RBE) in 16 fractions.^26^ Trismus was evaluated based on the CTCAE. Significant differences were observed in the Dmax values delivered to various temporomandibular joint‐related structures between trismus‐positive and trismus‐negative cases. Furthermore, ROC analysis revealed Dmax cut‐off values for the onset of trismus in these structures. The Dmax cut‐off values for the masseter muscle, temporal muscle, medial pterygoid muscle, lateral pterygoid muscle, and coronoid process were 44.0, 39.6, 60.4, 57.6, and 38.0 Gy (RBE), respectively. The areas under the curve (AUCs) for all structures ranged between 0.559 and 0.773, with the highest AUC corresponding to the coronoid process. The AUCs are low and indicate poor discrimination. This could be due to the small number of patients. Further studies are needed to verify these cut‐off values. Moreover, significant differences were found in the D10–50 indices for the coronoid process and in the D10 of the temporal muscle between trismus‐positive and trismus‐negative cases.

The study points out that the amount of dose incident to the coronoid process is unlikely to relate to trismus development. Therefore, it was suggested to use the Dmax of the masseter muscle (Dmax < 44.0 Gy [RBE]), which had the second highest AUC after the Dmax of the coronoid process, and the D10–50 of the coronoid process (D10–50 < 47.0 Gy [RBE]) as constraints. The coronoid process Dmax < 38.0 Gy (RBE) could be used as a reference during treatment planning.

## 9 MAXILLA, MANDIBLE, AND TEETH

Excess irradiation of the maxilla, mandible, and teeth may lead to various complications that reduce QOL, such as osteoradionecrosis (ORN), oronasal fistula, and tooth loss.^27^, ^28^, ^29^, ^30^ ORN causes difficulty in eating and maintaining oral hygiene.^27^ The oronasal fistula is a late sequela of the maxilla ORN.^28^ It causes nasal regurgitation of food, hypernasality of the voice, and taste disturbances.^28^ Similar to ORN, tooth loss causes masticatory dysfunction, swallowing dysfunction, and changes in appearance.^29^

ORN development after CIRT appears more in the oral cavity regions than in the HN regions.^27^ Previous studies have reported correlations between ORN development and maxilla or mandible irradiation.^27^, ^30^ Sasahara et al. investigated the correlation between ORN incidence and maxilla irradiation in 63 patients.^30^ The patients were treated with a total dose of 57.6 Gy (RBE) in 16 fractions. ORN was evaluated based on the CTCAE. Univariate analysis showed that the V10–50 indices significantly correlate with ORN development. However, multivariate analysis revealed that only V50 (V50 ≥ 3.0 cm^3^) is a significant independent risk factor for ORN. The 5‐year ORN occurrence rate for V50 ≥ 3.0 cm^3^ and V50 < 3.0 cm^3^ were 69.5% and 11.7%, respectively. Furthermore, the presence of teeth within the PTV was also found to be a significant risk factor.

Musha et al. focused on mandible irradiation during CIRT for 11 patients with non‐SCCs located in the oropharynx and floor of the mouth.^27^ The patients were treated with a total dose of 57.6 or 64 Gy (RBE) in 16 fractions. ORN occurrence was graded based on the CTCAE. ORN developed in patients who were irradiated with a large volume in the 30–40 Gy (RBE) dose region to the mandible and 10–25 Gy (RBE) dose region to the teeth. ROC analysis revealed that the use of mandible and teeth DVH parameters has high diagnostic performance. The cut‐off vales for the mandible parameters were V30 ≥ 16.5 cm^3^, V35 ≥ 16.4 cm^3^, V40 ≥ 12.9 cm^3^, V45 ≥ 8.3 cm^3^, and Dmean ≥ 22.8 Gy (RBE). The cut‐off values for the teeth parameters were V10 ≥ 3.4 cm^3^, V15 ≥ 3.0 cm^3^, V20 ≥ 2.5 cm^3^, V25 ≥ 2.1 cm^3^, V30 ≥ 1.8 cm^3^, and Dmean ≥ 22.7 Gy (RBE). Based on their level of significance, this study suggests the use of mandible V30 < 16.5 cm^3^ and teeth V30 < 1.8 cm^3^ as constraints. Moreover, contrary to Sasahara et al.,^30^ the results of Musha et al.^27^ suggest that the presence of teeth in the irradiation field is not a risk factor for ORN occurrence.

Bhattacharyya et al. investigated the development of oronasal fistula in 62 patients with sinonasal and oral cavity tumors, which were treated with 57.6 or 64 Gy (RBE) in 16 fractions.^28^ The severity of the oronasal fistula was evaluated using the CTCAE. Multivariate analysis revealed that both the use of chemotherapy and the number of teeth irradiated with a dose greater than 50 Gy (RBE) regardless of maxillary invasion are significant risk factors. In case 50 Gy (RBE) was incident to 2, 3, 4, or >5 teeth in a patient, the proportion of oronasal fistula development was 33%, 66%, 75%, and 100%, respectively.

A different study focused on the incidence of tooth loss after CIRT of 14 patients (171 teeth) with HN non‐SCCs.^29^ The patients were treated with 57.6 or 64 Gy (RBE) in 16 fractions. Cut‐off values for tooth loss occurrence were determined for the V10–60 indices using ROC analysis. These values were V10 ≥ 99.9%, V20 ≥ 99.1%, V30 ≥ 97.8%, V40 ≥ 78.1%, V50 ≥ 58.1%, and V60 ≥ 7.67%. More than half of the teeth that exceeded the V30–V60 cut‐off values fell out within 5 years. Kubo et al. recommend the use of V50 < 58.1% as a constraint because V50 showed the highest diagnostic performance among the DVH parameters.^29^

## 10 SKIN

A common side effect of HN cancer radiotherapy is acute radiation dermatitis (ARD).^31^ Li et al. investigated which prognostic factors are associated with ARD in 187 patients treated with a total dose of 57.6, 64, or 70.4 Gy (RBE) in 16 fractions.^31^ ARD was evaluated according to the CTCAE. The study constructed logistic regression models of grade 2–3 ARD NTCP for low‐dose (5–15 Gy [RBE]), middle‐dose (20–35 Gy [RBE]), and high‐dose (40–50 Gy [RBE]) regions. The skin surface area irradiated with at least 40 Gy (RBE) (S40) from the high‐dose group produced the NTCP model with the best performance. This agrees with the findings of a previous study that explored prognostic factors for acute skin reactions in malignant bone and soft tumor CIRT.^37^ The study showed that S40 is a significant predictive factor and suggested the use of S40 < 25 cm^2^ as a constraint.^37^ Therefore, it is important to aim in reducing the S40 during treatment planning. Li et al. recommend setting a value for the S40 constraint by considering the risk of ARD development during treatment planning based on the NTCP models reported.^31^

Table 1 summarizes the dose–volume constraints recommended by previous studies.^2^, ^17^, ^18^, ^19^, ^20^, ^21^, ^22^, ^23^, ^24^, ^25^, ^26^, ^27^, ^28^, ^29^, ^30^, ^31^ We divided the constraints into hard and soft. This will help in deciding which constraints to prioritize or whether more weight should be given to tumor coverage or OAR sparing during optimization if necessary.

**TABLE 1:** Dose–volume constraints suggested for clinical use

Organ	Parameter	Constraint	Total dose (Gy [RBE])	Number of fractions	Clinical endpoint	Hard/soft constraint	Study reference
Brain	V50 D5cm^3^	<4.6 cm^3^ <55.4 Gy (RBE) (TD5) <68.4 Gy (RBE) (TD50)	48–60.8 70.4	16 32	Grade ≥2 RIBI Grade ≥2 RIBI	Soft	Koto et al. (2014)^17^ Park et al. (2021)^19^
Brainstem	V30 V40	<0.7 cm^3^ <0.1 cm^3^	57.6–70.4	16	Grade 1 brainstem necrosis	Hard	Shirai et al. (2017)^2^
Parotid	V5	<41%	57.6–64	16	Parotid gland atrophy	Soft	Morikawa et al. (2016)^20^
Optic nerve	Dmax D20%	<57 Gy (RBE) <60 Gy (RBE) (TD50)	48–64	16–18	Visual loss	Hard	Hasegawa et al. (2006)^21^
Eyeball	Dmax V40	<54.75 Gy (RBE) <0.83 cm^3^	57.6–70.4	16	IOH/VH (grade 4 radiation retinopathy)	Hard	Nachankar et al. (2022)^22^
Retina	Dmax V40	<54.58 Gy (RBE) <0.66 cm^3^	57.6–70.4	16	IOH/VH (grade 4 radiation retinopathy)	Hard	Nachankar et al. (2022)^22^
Iris‐ciliary body	V50	<0.127 cm^3^	60–85	5	Neovascular glaucoma	Hard	Hirasawa et al. (2007)^23^
Optic disk	D50%	<50 Gy (RBE)	60–85	5	Neovascular glaucoma	Hard	Hirasawa et al. (2007)^23^
Nasolacrimal duct	V40	<0.08 cm^3^	57.6–64	16	Grade ≥1 nasolacrimal duct obstruction	Soft	Kubo et al. (2019)^24^
Tongue	Dmax	<54.3 Gy (RBE)	57.6–70.4	16	Grade ≥2 ARM	Soft	Musha et al. (2015)^25^
Palate	Dmax	<43.0 Gy (RBE)	57.6–70.4	16	Grade ≥2 ARM	Soft	Musha et al. (2015)^25^
Masseter muscle	Dmax	<44.0 Gy (RBE)	57.6–64	16	Grade 2 radiation‐induced trismus	Soft	Musha et al. (2020)^26^
Coronoid process	D10–50%	<47.0 Gy (RBE)	57.6–64	16	Grade 2 radiation‐induced trismus	Soft	Musha et al. (2020)^26^
Maxilla	V50	<3.0 cm^3^	57.6	16	ORN	Soft	Sasahara et al. (2014)^30^
Mandible	V30	<16.5 cm^3^	57.6–64	16	ORN	Soft	Musha et al. (2021)^27^
Teeth	V30 Number of teeth irradiated with *≥*50 Gy (RBE) V50	<1.8 cm^3^ ≤2 <58.1%	57.6–64 57.6–64 57.6–64	16 16 16	ORN Oronasal fistula Tooth loss	Soft	Musha et al. (2021)^27^ Bhattacharyya et al. (2020)^28^ Kubo et al. (2021)^29^
Skin	S40	Determine based on NTCP models reported	57.6–70.4	16	ARD	Soft	Li et al. (2022)^31^

Abbreviations: ARM, acute radiation mucositis; Dmax, maximum dose; Dxx, dose incident to xx cm^3^/% volume of organ at risk; IOH/VH, intraocular hemorrhage/vitreous hemorrhage; NTCP, normal tissue complication probability; ORN, osteoradionecrosis; RIBI, radiation‐induced brain injury; S40, surface area receiving a dose of 40 Gy (RBE); TDxx, tolerance dose corresponding to xx% probability of developing a complication; Vxx, volume of organ at risk receiving a dose of xx Gy (RBE).

## 11 DISCUSSION

CIRT has shown promising results in cancer treatment. However, considering that patients tend to survive for a long time after treatment, complications such as radiation‐induced brainstem necrosis, RIBI, visual loss, radiation‐induced trismus, and ORN are considered to be critical adverse effects because they affect the patient's QOL and can potentially be life‐threatening.^2^, ^17^, ^18^, ^19^, ^21^, ^22^, ^26^, ^27^, ^28^, ^29^, ^30^ Furthermore, acute complications, such as ARM and ARD, discourage patients from participating in treatment.^25^, ^31^ Establishment of dose–volume constraints will play an important role in assisting treatment plan optimization, ensuring complications are minimized, enabling multicenter studies and comparisons between different modalities, and facilitating adaptive radiotherapy. At each fraction, it will be possible to confirm whether replanning is necessary based on the constraints and OAR irradiation. This review summarizes the findings of previous research with regard to DVH parameters associated with adverse effects in HN cancer CIRT.

In radiotherapy, particularly for advanced treatment techniques such as IMRT, volumetric‐modulated arc therapy, and intensity‐modulated proton therapy (IMPT), hard and soft constraints are used.^38^ Hard constraints are constraints that in principle are not allowed to be violated, while soft constraints should be achieved as well as possible even though they can be violated. Constraints should be treated as hard and soft in CIRT, as well, especially for institutions that plan to use intensity‐modulated carbon therapy or adaptive radiotherapy. We divided the reported constraints into hard and soft (Table 1). The distinction was based on various factors, including the severity of the complication, and whether there are methods to prevent the occurrence of a complication or treat the symptom. For example, the brainstem constraints were set as hard because radiation‐induced brainstem necrosis would affect essential functions such as respiratory motion.^2^ Similarly, the constraints for the optic nerve, eyeball, retina, iris‐ciliary body, and optic disk were set as hard because complications in these OARs may lead to visual loss.^21^, ^22^, ^23^ If excess irradiation cannot be prevented because the tumor is in close proximity, the constraints of the unaffected respective OAR should definitely be met. On the other hand, constraints for OARs like the nasolacrimal duct and tongue were set as soft since nasolacrimal duct obstruction can be treated with dacryocystorhinostomy, and complications to the tongue can be reduced by using a mouthpiece.^39^, ^40^

Various strategies have been proposed for reducing the dose delivered to OARs and consequently contribute to fulfilling dose–volume constraints.^28^, ^31^, ^40^, ^41^ Musha et al. used customized mouthpieces in patients with tumors of the nasal and paranasal sinuses to reduce the dose delivered to the tongue.^40^ The mouthpieces were created to displace the tongue to low‐dose regions that were identified using a dose surface model.^25^ Such mouthpieces should be close‐fitting to fix the position of the teeth of both jaws and maintain the position of the lower jaw, not cause discomfort, and reduce mucositis.^40^ For sinonasal and oral cavity tumors, the probability of oronasal fistula occurrence may be reduced by considering the high‐dose region near the teeth during contouring of the clinical target volume or by minimizing PTV margins.^28^ Moreover, compared with the broad‐beam technique, using the layer stacking technique in passive irradiation can improve skin sparing and reduce grade 2–3 ARD occurrence.^31^, ^41^ However, for tumors that have a diameter less than 30 mm or are close to the skin, layer stacking may provide no clinical benefit. Pencil‐beam scanning (PBS) irradiation is another delivery technique that may reduce the dose of OARs. Contrary to passive irradiation techniques, PBS irradiation has better dose conformity.^42^ Implementing advanced techniques that use PBS irradiation in CIRT, like IMPT, will further improve OAR sparing. However, particle therapy for HN cancer using PBS is highly susceptible to range uncertainties arising from the conversion of the Hounsfield units of computed tomography images to relative stopping power values and image artifacts.^43^ Also, it is sensitive to setup errors and anatomical variations due to tumor response and changes in the weight of the patient.^44^

Some of the complications considered in this review differ from those in XRT. CIRT in Japan is generally indicated for non‐SCCs mainly located at the nasal, paranasal, and maxillary sinus sites, while tumors like the SCCs of the oral cavity, larynx, and pharynx are treated with XRT because they are radiosensitive. Therefore, complications such as laryngeal and pharyngeal dysfunction do not occur after HN cancer CIRT, and, therefore, their respective constraints are not included in our list of constraints.

The clinical experience in CIRT is limited; only Japan and Germany have extensive experience. Therefore, CIRT institutions can benefit from using the long‐term clinical data of Japanese and German institutions. The constraints summarized in this review are directly applicable to Japanese facilities. LEM‐based CIRT institutions that wish to benefit from the Japanese data, including prescription doses and our list of constraints, will have to apply conversion factors for biological dose conversion.^45^, ^46^ Previously reported conversion factors or curves depend on various parameters, including the SOBP width and depth, fractionation, tissue characteristics, and endpoint.^15^, ^42^ Therefore, when using conversion factors, the specific parameters used to derive the factors need to be considered. Even if the dose is corrected, there might be physical dose variations due to different clinical dose optimization algorithms and delivery techniques.^46^ Current studies on the conversion of Japanese constraints to LEM‐based constraints are limited.^15^, ^46^, ^47^, ^48^, ^49^, ^50^, ^51^, ^52^ One study produced a dose translation model to convert brainstem constraints from the Japanese model to the LEM. Japanese and LEM‐based treatment plans were designed. For each brainstem, the Japanese constraints were plotted against the corresponding LEM‐based constraints and a curve‐fitting procedure was performed to produce the dose translation model.^16^ Another study used isodose volumes in Japanese and LEM‐based plans to define conversion factors for various doses and use these to translate Japanese constraints of the rectum to the corresponding LEM‐based constraints.^47^ Future studies should aim to determine conversion factors for a wide range of tumor sites and OARs. Converting Japanese prescription doses and OAR constraints to the LEM would facilitate comparisons between Japanese institutions and other institutions worldwide leading to the improvement of the CIRT implementation.

The majority of the studies included in this review were conducted using a small number of patients. This indicates that their results might not be reliable. Selecting constraints based solely on indications such as the AUC or significance (*p*‐values) may result in ignoring constraints that are in reality more powerful in predicting toxicity development than those selected. Table S1 shows all dose–volume constraints that were found to be related to toxicity occurrence before determining significant risk factors. This table could be treated as a supplementary reference guide if necessary. Studies with a sufficiently large number of patients are necessary to confirm the reliability of the constraints reported.

To ensure the applicability of the dose–volume constraints, uncertainties in the absorbed doses and LET distributions in the OARs should be considered. The uncertainty in the absorbed doses in the target region was reported to vary by about 2.5% for HN cancer CIRT.^16^ However, the variations in the absorbed doses in the out‐of‐target region have not been determined, but are expected to be larger than 2.5%, particularly within the lateral penumbra dose fall‐off.^31^ This will have a direct impact on the dose delivered to the OARs close to the target. Skin dose has also been shown to vary about 4% due to potential electron fluence and secondary particles arising from the particle interactions in the snout and the scattering material in the beam path.^53^, ^54^ Dose variations due to these effects seem to be more pronounced in passive particle radiotherapy.^53^ Accurate techniques for estimating the dose delivered to the skin should be employed.^31^, ^55^, ^56^ Furthermore, the linear energy transfer (LET) distribution was not considered in any study. High LET radiation is associated with increased radiation‐induced toxicity.^57^ Considering the LET distribution inside the OAR region has the potential of providing further information on toxicity development. Additionally, RBE values for OARs should be considered, especially for those that are close to the tumor and may fall into high LET volumes. Unfortunately, reported data are limited and focused on animal studies.^58^

The list of the dose–volume constraints reported is not thorough. The constraints reported for RIBI, parotid gland atrophy, and ARM are insufficient and incomplete.^17^, ^20^, ^25^ Constraints for the low‐dose regions of the brain, a definite value for the V5 constraint for the parotid, and constraints for the oral mucosa need to be determined. Additionally, it is necessary to determine constraints for preventing toxicities that also impact patient QOL, such as acute parotitis and dysgeusia development. Finally, future studies should focus on developing NTCP curves for all toxicities associated with CIRT.

## 12 CONCLUSIONS

Currently, there are no established standard dose–volume constraints for CIRT. This review is the first step in establishing constraints for HN cancer CIRT. Establishing constraints in CIRT will aid in increasing the safety and efficacy aspects of CIRT. The review presents a list of dose–volume constraints for HN cancer CIRT based on the Japanese RBE models. These constraints can serve as a guide during treatment planning to ensure toxicities are kept at a minimum. The list of constraints should keep being updated based on the results of new studies. Various planning strategies are also presented, which can be used to reduce irradiation to the OARs.

## AUTHOR CONTRIBUTIONS

**Maria Varnava:** Conceptualization (equal); investigation (equal); methodology (lead); resources (equal); writing – original draft (lead); writing – review and editing (equal). **Atsushi Musha:** Conceptualization (equal); resources (supporting); writing – review and editing (equal). **Mutsumi Tashiro:** Resources (supporting); writing – review and editing (equal). **Nobuteru Kubo:** Writing – review and editing (equal). **Naoko Okano:** Writing – review and editing (equal). **Hidemasa Kawamura:** Writing – review and editing (equal). **Tatsuya Ohno:** Conceptualization (lead); investigation (equal); resources (equal); writing – review and editing (equal).

## FUNDING INFORMATION

None

## CONFLICT OF INTEREST

NO and TO report research grants from Hitachi.

## Supporting information


Table S1.

## Data Availability

Not applicable.

## References

[1] Chow LQM . Head and neck cancer. N Engl J Med. 2020;382(1):60‐72. doi:10.1056/NEJMra1715715 31893516

[2] Shirai K , Fukata K , Adachi A , et al. Dose‐volume histogram analysis of brainstem necrosis in head and neck tumors treated using carbon‐ion radiotherapy. Radiother Oncol. 2017;125(1):36‐40. doi:10.1016/j.radonc.2017.08.014 28867558

[3] Okada T , Kamada T , Tsuji H , et al. Carbon ion radiotherapy: clinical experiences at National Institute of radiological science (NIRS). J Radiat Res. 2010;51(4):355‐364. doi:10.1269/jrr.10016 20508375

[4] Mohamad O , Sishc BJ , Saha J , et al. Carbon ion radiotherapy: a review of clinical experiences and preclinical research, with an emphasis on DNA damage/repair. Cancer. 2017;9(6):66. doi:10.3390/cancers9060066 PMC5483885 28598362

[5] Saitoh JI , Koto M , Demizu Y , et al. A multicenter study of carbon‐ion radiation therapy for head and neck adenocarcinoma. Int J Radiat Oncol Biol Phys. 2017;99(2):442‐449. doi:10.1016/j.ijrobp.2017.04.032 28871995

[6] Emami B , Lyman J , Brown A , et al. Tolerance of normal tissue to therapeutic irradiation. Int J Radiat Oncol Biol Phys. 1991;21(1):109‐122. doi:10.1016/0360-3016(91)90171-y 2032882

[7] Bentzen SM , Constine LS , Deasy JO , et al. Quantitative analyses of Normal tissue effects in the clinic (QUANTEC): an introduction to the scientific issues. Int J Radiat Oncol Biol Phys. 2010;76(3 Suppl):S3‐S9. doi:10.1016/j.ijrobp.2009.09.040 20171515 PMC3431964

[8] Brodin NP , Tomé WA . Revisiting the dose constraints for head and neck OARs in the current era of IMRT. Oral Oncol. 2018;86:8‐18. doi:10.1016/j.oraloncology.2018.08.018 30409324 PMC6481934

[9] International Commission on Radiation Units and Measurements . ICRU report 93: prescribing, recording, and reporting light ion beam therapy. J ICRU. 2016;16:1‐211.

[10] International Commission on Radiation Units and Measurements . ICRU report 78: prescribing, recording, and reporting proton beam therapy. J ICRU. 2007;7:NP.

[11] Fossati P , Perpar A , Stock M , et al. Carbon ion dose constraints in the head and neck and skull base: review of MedAustron institutional protocols. Int J Part Ther. 2021;8(1):25‐35. doi:10.14338/IJPT-20-00093.1 PMC8270085 34285933

[12] Kanai T , Furusawa Y , Fukutsu K , Itsukaichi H , Eguchi‐Kasai K , Ohara H . Irradiation of mixed beam and design of spread‐out Bragg peak for heavy‐ion radiotherapy. Radiat Res. 1997;147(1):78‐85. doi:10.2307/3579446 8989373

[13] Inaniwa T , Furukawa T , Kase Y , et al. Treatment planning for a scanned carbon beam with a modified microdosimetric kinetic model. Phys Med Biol. 2010;55(22):6721‐6737. doi:10.1088/0031-9155/55/22/008 21030747

[14] Fossati P , Matsufuji N , Kamada T , Karger CP . Radiobiological issues in prospective carbon ion therapy trials. Med Phys. 2018;45(11):e1096‐e1110. doi:10.1002/mp.12506 30421806

[15] Wang W , Huang Z , Sheng Y , et al. RBE‐weighted dose conversions for carbon ion radiotherapy between microdosimetric kinetic model and local effect model for the targets and organs at risk in prostate carcinoma. Radiother Oncol. 2020;144:30‐36. doi:10.1016/j.radonc.2019.10.005 31710941

[16] Dale JE , Molinelli S , Vischioni B , et al. Brainstem NTCP and dose constraints for carbon ion RT‐application and translation from Japanese to European RBE‐weighted dose. Front Oncol. 2020;10:531344. doi:10.3389/fonc.2020.531344 33330020 PMC7735105

[17] Koto M , Hasegawa A , Takagi R , et al. Risk factors for brain injury after carbon ion radiotherapy for skull base tumors. Radiother Oncol. 2014;111(1):25‐29. doi:10.1016/j.radonc.2013.11.005 24332023

[18] Kishimoto R , Mizoe JE , Komatsu S , Kandatsu S , Obata T , Tsujii H . MR imaging of brain injury induced by carbon ion radiotherapy for head and neck tumors. Magn Reson Med Sci. 2005;4(4):159‐164. doi:10.2463/mrms.4.159 16543700

[19] Park S , Demizu Y , Suga M , et al. Predicted probabilities of brain injury after carbon ion radiotherapy for head and neck and skull base tumors in long‐term survivors. Radiother Oncol. 2021;165:152‐158. doi:10.1016/j.radonc.2021.10.017 34718054

[20] Morikawa T , Koto M , Hasegawa A , et al. Radiation‐induced parotid gland atrophy in patients with head and neck cancer after carbon‐ion radiotherapy. Anticancer Res. 2016;36(10):5403‐5407. doi:10.21873/anticanres.11116 27798906

[21] Hasegawa A , Mizoe JE , Mizota A , Tsujii H . Outcomes of visual acuity in carbon ion radiotherapy: analysis of dose‐volume histograms and prognostic factors. Int J Radiat Oncol Biol Phys. 2006;64(2):396‐401. doi:10.1016/j.ijrobp.2005.07.298 16182466

[22] Nachankar A , Musha A , Kubo N , et al. Dosimetric analysis of intraocular hemorrhage in nonsquamous head and neck cancers treated with carbon‐ion radiotherapy. Radiother Oncol. 2022;170:143‐150. doi:10.1016/j.radonc.2022.02.032 35257851

[23] Hirasawa N , Tsuji H , Ishikawa H , et al. Risk factors for neovascular glaucoma after carbon ion radiotherapy of choroidal melanoma using dose‐volume histogram analysis. Int J Radiat Oncol Biol Phys. 2007;67(2):538‐543. doi:10.1016/j.ijrobp.2006.08.080 17141971

[24] Kubo N , Kubota Y , Kawamura H , et al. Dosimetric parameters predictive of nasolacrimal duct obstruction after carbon‐ion radiotherapy for head and neck carcinoma. Radiother Oncol. 2019;141:72‐77. doi:10.1016/j.radonc.2019.07.022 31439449

[25] Musha A , Shimada H , Shirai K , et al. Prediction of acute radiation mucositis using an oral mucosal dose surface model in carbon ion radiotherapy for head and neck tumors. PLoS One. 2015;10(10):e0141734. doi:10.1371/journal.pone.0141734 26512725 PMC4626117

[26] Musha A , Shimada H , Kubo N , et al. Evaluation of carbon ion radiation‐induced trismus in head and neck tumors using dose‐volume histograms. Cancer. 2020;12(11):3116. 10.3390/cancers12113116 PMC7693287 33113829

[27] Musha A , Shimada H , Kubo N , et al. Clinical features and dosimetric evaluation of carbon ion radiation‐induced osteoradionecrosis of mandible in head and neck tumors. Radiother Oncol. 2021;161:205‐210. doi:10.3390/cancers12113116 34147522

[28] Bhattacharyya T , Koto M , Ikawa H , Hayashi K , Hagiwara Y , Tsuji H . Assessment of risk factors associated with development of oronasal fistula as a late complication after carbon‐ion radiotherapy for head and neck cancer. Radiother Oncol. 2020;144:53‐58. doi:10.1016/j.radonc.2019.10.015 31733488

[29] Kubo N , Sakai M , Kawamura H , et al. Dosimetric parameters predicting tooth loss after carbon ion radiotherapy for head and neck tumors. Radiat Ther. 2021;1(3):183‐193. doi:10.3390/radiation1030017

[30] Sasahara G , Koto M , Ikawa H , et al. Effects of the dose‐volume relationship on and risk factors for maxillary osteoradionecrosis after carbon ion radiotherapy. Radiat Oncol. 2014;9(1):92. doi:10.1186/1748-717X-9-92 24708583 PMC3992144

[31] Li Y , Sakai M , Tsunoda A , et al. Normal tissue complication probability model for acute radiation dermatitis in patients with head and neck cancer treated with carbon ion radiation therapy. Int J Radiat Oncol Biol Phys. 2022;113(3):675‐684. doi:10.1016/j.ijrobp.2022.03.002 35278673

[32] Kanai T , Endo M , Minohara S , et al. Biophysical characteristics of HIMAC clinical irradiation system for heavy‐ion radiation therapy. Int J Radiat Oncol Biol Phys. 1999;44(1):201‐210. doi:10.1016/s0360-3016(98)00544-6 10219815

[33] Eisbruch A , Ten Haken RK , Kim HM , Marsh LH , Ship JA . Dose, volume, and function relationships in parotid salivary glands following conformal and intensity‐modulated irradiation of head and neck cancer. Int J Radiat Oncol Biol Phys. 1999;45(3):577‐587. doi:10.1016/s0360-3016(99)00247-3 10524409

[34] Maria OM , Eliopoulos N , Muanza T . Radiation‐induced oral mucositis. Front Oncol. 2017;7:89. doi:10.3389/fonc.2017.00089 28589080 PMC5439125

[35] Singh K , Rashmikant US , Alvi HA , Singh RK . Management of trismus following radiation therapy by cost‐effective approach. BMJ Case Rep. 2012;2012:bcr2012007326. doi:10.1136/bcr-2012-007326 PMC4543707 23087289

[36] Teguh DN , Levendag PC , Voet P , et al. Trismus in patients with oropharyngeal cancer: relationship with dose in structures of mastication apparatus. Head Neck. 2008;30(5):622‐630. doi:10.1002/hed.20760 18213726

[37] Takakusagi Y , Saitoh JI , Kiyohara H , et al. Predictive factors of acute skin reactions to carbon ion radiotherapy for the treatment of malignant bone and soft tissue tumors. Radiat Oncol. 2017;12(1):185. doi:10.1186/s13014-017-0927-4 29166945 PMC5700693

[38] Hansen CR , Crijns W , Hussein M , et al. Radiotherapy treatment plannINg study guidelines (RATING): a framework for setting up and reporting on scientific treatment planning studies. Radiother Oncol. 2020;153:67‐78. doi:10.1016/j.radonc.2020.09.033 32976873

[39] El‐Sawy T , Ali R , Nasser QJ , Esmaeli B . Outcomes of dacryocystorhinostomy in patients with head and neck cancer treated with high‐dose radiation therapy. Ophthal Plast Reconstr Surg. 2012;28(3):196‐198. doi:10.1097/IOP.0b013e31824c11df PMC3878067 22460683

[40] Musha A , Saitoh J , Shirai K , et al. Customized mouthpieces designed to reduce tongue mucositis in carbon‐ion radiotherapy for tumors of the nasal and paranasal sinuses. Phys Imaging Radiat Oncol. 2017;3:1‐4. doi:10.1016/j.phro.2017.07.003

[41] Kubo N , Kubota Y , Oike T , et al. Skin dose reduction by layer‐stacking irradiation in carbon ion radiotherapy for parotid tumors. Front Oncol. 2020;10:1396. doi:10.3389/fonc.2020.01396 32923391 PMC7456805

[42] Mohamad O , Makishima H , Kamada T . Evolution of carbon ion radiotherapy at the National Institute of Radiological Sciences in Japan. Cancer. 2018;10(3):66. doi:10.3390/cancers10030066 PMC5876641 29509684

[43] Malyapa R , Lowe M , Bolsi A , Lomax AJ , Weber DC , Albertini F . Evaluation of robustness to setup and range uncertainties for head and neck patients treated with pencil beam scanning proton therapy. Int J Radiat Oncol Biol Phys. 2016;95(1):154‐162. doi:10.1016/j.ijrobp.2016.02.016 27084638

[44] Mohamed N , Lee A , Lee NY . Proton beam radiation therapy treatment for head and neck cancer. Precis Radiat Oncol. 2022;6(1):59‐68. doi:10.1002/pro6.1135

[45] Steinsträter O , Grün R , Scholz U , Friedrich T , Durante M , Scholz M . Mapping of RBE‐weighted doses between HIMAC‐ and LEM‐based treatment planning systems for carbon ion therapy. Int J Radiat Oncol Biol Phys. 2012;84(3):854‐860. doi:10.1016/j.ijrobp.2012.01.038 22483698

[46] Molinelli S , Magro G , Mairani A , et al. Dose prescription in carbon ion radiotherapy: how to compare two different RBE‐weighted dose calculation systems. Radiother Oncol. 2016;120(2):307‐312. doi:10.1016/j.radonc.2016.05.031 27394694

[47] Wang W , Li P , Sheng Y , et al. Conversion and validation of rectal constraints for prostate carcinoma receiving hypofractionated carbon‐ion radiotherapy with a local effect model. Radiat Oncol. 2021;16(1):72. doi:10.1186/s13014-021-01801-w 33849589 PMC8045205

[48] Dale JE , Molinelli S , Vitolo V , et al. Optic nerve constraints for carbon ion RT at CNAO ‐ reporting and relating outcome to European and Japanese RBE. Radiother Oncol. 2019;140:175‐181. doi:10.1016/j.radonc.2019.06.028 31310888

[49] Choi K , Molinelli S , Russo S , et al. Rectum dose constraints for carbon ion therapy: relative biological effectiveness model dependence in relation to clinical outcomes. Cancer. 2019;12(1):46. doi:10.3390/cancers12010046 PMC7016830 31877802

[50] Vischioni B , Russo S , Meuli M , et al. Dosimetric and clinical risk factors for the development of maxillary osteoradionecrosis in adenoid cystic carcinoma (ACC) patients treated with carbon ion radiotherapy. Front Oncol. 2022;12:829502. doi:10.3389/fonc.2022.829502 35311095 PMC8924362

[51] Molinelli S , Bonora M , Magro G , et al. RBE‐weighted dose in carbon ion therapy for ACC patients: impact of the RBE model translation on treatment outcomes. Radiother Oncol. 2019;141:227‐233. doi:10.1016/j.radonc.2019.08.022 31522881

[52] Grosshagauer S , Fossati P , Schafasand M , et al. Organs at risk dose constraints in carbon ion radiotherapy at MedAustron: translations between LEM and MKM RBE models and preliminary clinical results. Radiother Oncol. 2022;175:73‐78. doi:10.1016/j.radonc.2022.08.008 35952977

[53] Yonai S , Matsufuji N , Kanai T , et al. Measurement of neutron ambient dose equivalent in passive carbon‐ion and proton radiotherapies. Med Phys. 2008;35(11):4782‐4792. doi:10.1118/1.2989019 19070210

[54] Kern A , Bäumer C , Kröninger K , Wulff J , Timmermann B . Impact of air gap, range shifter, and delivery technique on skin dose in proton therapy. Med Phys. 2021;48(2):831‐840. doi:10.1002/mp.14626 33368345

[55] Arbor N , Gasteuil J , Noblet C , Moreau M , Meyer P . A GATE/Geant4 Monte Carlo toolkit for surface dose calculation in VMAT breast cancer radiotherapy. Phys Med. 2019;61:112‐117. doi:10.1016/j.ejmp.2019.04.012 31036441

[56] Schreuder AN , Bridges DS , Rigsby L , et al. Validation of the RayStation Monte Carlo dose calculation algorithm using realistic animal tissue phantoms. J Appl Clin Med Phys. 2019;20(10):160‐171. doi:10.1002/acm2.12733 PMC6806482 31541536

[57] Bassler N , Toftegaard J , Lühr A , et al. LET‐painting increases tumour control probability in hypoxic tumours. Acta Oncol. 2014;53(1):25‐32. doi:10.3109/0284186X.2013.832835 24020629

[58] Karger CP , Peschke P . RBE and related modeling in carbon‐ion therapy. Phys Med Biol. 2017;63(1):01TR02. doi:10.1088/1361-6560/aa9102 28976361

