# Supplementary material for: Dose–volume constraints for head‐and‐neck cancer in carbon ion radiotherapy: A literature review
Source: Cancer Med. 2023 Feb 17;12(7):8267–77. doi: 10.1002/cam4.5641 (PMC10134371; doi:10.1002/cam4.5641)
Supplement: Supplementary file 1 — Table S1. [file CAM4-12-8267-s001.docx]

**Supplementary Material**

Supplementary Table S1. All dose–volume constraints that were initially considered to be significant predictors of adverse effects prior to using a selection method.

| **Organ** | **Parameter** | **Constraint** | **Total dose (Gy [RBE])** | **Number of fractions** | **Clinical endpoint** | **Selection method** | **Study reference** |
| --- | --- | --- | --- | --- | --- | --- | --- |
| Brain | V40  **V50**^†^  Dmax  D1%  D2%  D1–4cm^3^  **D5cm^3^**^†^  D6–10cm^3^  V1–71 | <7.6 cm^3^  **<4.6 cm^3^**  -  -  -  -  **<55.4 Gy (RBE)**  **(TD5)**  **<68.4 Gy (RBE)**  **(TD50)**  -  - | 48–60.8  70.4 | 16  32 | Grade ≥ 2 RIBI  Grade ≥ 2 RIBI | Multivariate analysis  Akaike information criterion  +  Multivariate analysis | Koto et al.  (2014)^17^  Park et al.  (2021)^19^ |
| Brainstem | Dmax  D1cm^3^  V20  **V30**^†^  **V40**^†^ | <48 Gy (RBE)  <27 Gy (RBE)  <1.4 cm^3^  **<0.7 cm^3^**  **<0.1 cm^3^** | 57.6–70.4 | 16 | Grade 1 brain necrosis | Multivariate analysis | Shirai et al.  (2017)^2^ |
| Parotid | Dmean  Dmax  **V5**^†^  V10  V15  V20 | -  -  -  -  -  - | 57.6–64 | 16 | Parotid gland atrophy | Multivariate analysis | Morikawa et al. (2016)^20^ |
| Optic nerve | Dmax  D10%  **D20%**^†^  D30%  D40%  D50%  V10  V20  V30  V40  V50 | <57 Gy (RBE)  <63 Gy (RBE)  (TD50)  **<60 Gy (RBE)**  **(TD50)**  <59 Gy (RBE)  (TD50)  <51 Gy (RBE)  (TD50)  <51 Gy (RBE)  (TD50)  <0.7 cm^3^  <0.7 cm^3^  <0.7 cm^3^  <0.7 cm^3^  <0.53 cm^3^ | 48–64  57.6–70.4 | 16–18  16 | Visual loss  IOH/VH  (grade 4 radiation retinopathy) | Multivariate analysis  Clinical applicability | Hasegawa et al. (2006)^21^  Nachankar et al. (2022)^22^ |
| Eyeball | **Dmax**^†^  V10  V20  V30  **V40**^†^  V50  V60 | **<54.75 Gy (RBE)**  <4.84 cm^3^  <2.69 cm^3^  <2.29 cm^3^  *<***0.83 cm^3^**  <1.45 cm^3^  <0.45 cm^3^ | 57.6–70.4 | 16 | IOH/VH  (grade 4 radiation retinopathy) | Clinical applicability | Nachankar et al. (2022)^22^ |
| Retina | **Dmax**^†^  V10  V20  V30  **V40**^†^  V50  V60  V50 | **<54.58 Gy (RBE)**  <2.09 cm^3^  <1.82 cm^3^  <1.35 cm^3^  **<0.66 cm^3^**  <0.92 cm^3^  <0.35 cm^3^  <0.402 cm^3^ | 57.6–70.4  60–85 | 16  5 | IOH/VH  (grade 4 radiation retinopathy)  Neovascular glaucoma | Clinical applicability  Multivariate analysis | Nachankar et al. (2022)^22^  Hirasawa et al. (2007)^23^ |
| Iris-ciliary body | **V50**^†^ | **<0.127 cm^3^** | 60–85 | 5 | Neovascular glaucoma | Multivariate analysis | Hirasawa et al. (2007)^23^ |
| Optic disk | **D50%**^†^ | **<50 Gy (RBE)** | 60–85 | 5 | Neovascular glaucoma | Multivariate analysis | Hirasawa et al. (2007)^23^ |
| Nasolacrimal duct | V10  V20  V30  **V40**^†^  V50  V60 | <0.24 cm^3^  <0.24 cm^3^  <0.17 cm^3^  **<0.08 cm^3^**  <0.19 cm^3^  <0.04 cm^3^ | 57.6–64 | 16 | Grade ≥1 nasolacrimal duct obstruction | Clinical applicability  +  Univariate analysis | Kubo et al.  (2019)^24^ |
| Tongue | **Dmax**^†^ | **<54.3 Gy (RBE)** | 57.6–70.4 | 16 | Grade ≥ 2 ARM | - | Musha et al. (2015)^25^ |
| Palate | **Dmax**^†^ | **<43.0 Gy (RBE)** | 57.6–70.4 | 16 | Grade ≥ 2 ARM | - | Musha et al. (2015)^25^ |
| Masseter muscle | **Dmax**^†^ | **<44.0 Gy (RBE)** | 57.6–64 | 16 | Grade 2 radiation-induced trismus | AUC value | Musha et al. (2020)^26^ |
| Temporal muscle | Dmax  D10% | <39.6 Gy (RBE)  - | 57.6–64 | 16 | Grade 2 radiation-induced trismus | AUC value | Musha et al. (2020)^26^ |
| Medial pterygoid muscle | Dmax | <60.4 Gy (RBE) | 57.6–64 | 16 | Grade 2 radiation-induced trismus | AUC value | Musha et al. (2020)^26^ |
| Lateral pterygoid muscle | Dmax | <57.6 Gy (RBE) | 57.6–64 | 16 | Grade 2 radiation-induced trismus | AUC value | Musha et al. (2020)^26^ |
| Coronoid process | Dmax  **D10–50%**^†^ | <38.0 Gy (RBE)  **<47.0 Gy (RBE)** | 57.6–64 | 16 | Grade 2 radiation-induced trismus | Observations | Musha et al. (2020)^26^ |
| Maxilla | V10  V20  V30  V40  **V50**^†^ | <14.5 cm^3^  <11.9 cm^3^  <8.1 cm^3^  <4.6 cm^3^  **<3.0 cm^3^** | 57.6 | 16 | ORN | Multivariate analysis | Sasahara et al. (2014)^30^ |
| Mandible | Dmean  **V30**^†^  V35  V40  V45 | <22.8 Gy (RBE)  **<16.5 cm^3^**  <16.4 cm^3^  <12.9 cm^3^  <8.3 cm^3^ | 57.6–64 | 16 | ORN | Significance  (*p*-value) | Musha et al. (2021)^27^ |
| Teeth | Dmean  V10  V15  V20  V25  **V30**^†^  Number of teeth in the PTV / irradiated with ≥40 Gy (RBE)  **Number of teeth irradiated with ≥50 Gy (RBE)**^†^  V50  (without maxillary invasion)  D5cm^3^  (without maxillary invasion)  V10  V20  V30  V40  **V50**^†^  V60 | <22.7 Gy (RBE)  <3.4 cm^3^  <3.0 cm^3^  <2.5 cm^3^  <2.1 cm^3^  **<1.8 cm^3^**  ≤2  **≤2**  ≤6.6  ≤54.5 Gy (RBE)  <99.9%  <99.1%  <97.8%  <78.1%  **<58.1%**  <7.67% | 57.6–64  57.6–64  57.6–64 | 16  16  16 | ORN  Oronasal fistula  Tooth loss | Significance  (*p*-value)  Multivariate analysis  Accuracy | Musha et al. (2021)^27^  Bhattacharyya et al. (2020)^28^  Kubo et al.  (2021)^29^ |
| Skin | S15  S35  **S40**^†^ | Determine based on NTCP models reported | 57.6–64 | 16 | ARD | AUC  +  Akaike corrected information criterion values | Li et al.  (2022)^31^ |

Abbreviations: Vxx, volume of organ at risk receiving a dose of xx Gy (RBE); Dxx, dose incident to xx cm^3^/% volume of organ at risk; TDxx, tolerance dose corresponding to xx% probability of developing a complication; Dmax, maximum dose; Dmean, mean dose; Sxx, surface area receiving a dose of xx Gy (RBE); RIBI, radiation-induced brain injury; IOH/VH, intraocular hemorrhage/vitreous hemorrhage; ARM, acute radiation mucositis; ORN, osteoradionecrosis; ARD, acute radiation dermatitis; NTCP, normal tissue complication probability; AUC, area under the curve.

All parameters listed were determined to be significantly related to respective toxicities using statistical methods. The selection method refers to the method used to determine which parameters are significant risk factors.

^†^Parameters highlighted in bold correspond to the parameters determined by the selection method. These constraints are suggested to be used as constraints by previous research.
